# Imidazolium Ionic Liquid Modified Graphene Oxide: As a Reinforcing Filler and Catalyst in Epoxy Resin

**DOI:** 10.3390/polym9090447

**Published:** 2017-09-14

**Authors:** Qing Lyu, Hongxia Yan, Lin Li, Zhengyan Chen, Huanhuan Yao, Yufeng Nie

**Affiliations:** 1 Department of Applied Chemistry, School of Natural and Applied Sciences, Northwestern Polytechnical University, Xi’an 710129, China; lq429216099@live.com (Q.L.); lilin20172017@outlook.com (L.L.); chenzhengyan@mail.nwpu.edu.cn (Z.C.); huanhuanyao@outlook.com (H.Y.); 2 Department of Applied Mathematics, School of Natural and Applied Sciences, Northwestern Polytechnical University, Xi’an 710129, China; yfnie@nwpu.edu.cn

**Keywords:** graphene oxide, epoxy, ionic liquid, imidazole

## Abstract

Surface modification of graphene oxide (GO) is one of the most important issues to produce high performance GO/epoxy composites. In this paper, the imidazole ionic liquid (IMD-Si) was introduced onto the surface of GO sheets by a cheap and simple method, to prepare a reinforcing filler, as well as a catalyst in epoxy resin. The interlayer spacing of GO sheets was obviously increased by the intercalation of IMD-Si, which strongly facilitated the dispersibility of graphene oxide in organic solvents and epoxy matrix. The addition of 0.4 wt % imidazolium ionic liquid modified graphene oxide (IMD-Si@GO), yielded a 12% increase in flexural strength (141.3 MPa), a 26% increase in flexural modulus (4.69 GPa), and a 52% increase in impact strength (18.7 kJ/m^2^), compared to the neat epoxy. Additionally the IMD-Si@GO sheets could catalyze the curing reaction of epoxy resin-anhydride system significantly. Moreover, the improved thermal conductivities and thermal stabilities of epoxy composites filled with IMD-Si@GO were also demonstrated.

## 1. Introduction

As a kind of classic thermosetting polymers, epoxy resins are of particular interest to structural engineers due to their good stiffness, high strength, excellent dimensional stability, low curing shrinkage, and unique chemical resistance. Nevertheless, inherent brittleness strongly hinders the applications of epoxy resins in many fields. A large number of researchers have focused on the reinforcement of epoxy matrices with nano-fillers, such as SiO_2_, Al_2_O_3_, Fe_3_O_4_, carbon nanotubes, etc. [1,2,3,4]. As is well known, graphene, one of the most effective additives due to its exceptional physical properties, is widely used to improve the comprehensive performances of epoxy composites, such as mechanical properties, electrical properties and thermal properties [5]. However, the lack of reactive functional groups on the graphene surface inhibits the use of graphene, due to the poor dispersibility and extremely weak interfacial bonding between graphene and epoxy matrix [6].

Graphene oxide (GO), as an oxide form of graphene, contains a large number of oxygenated groups on the basal planes and along the edges. As a result, GO has high organic compatibility with matrices and can be a suitable nano-filler to reinforce epoxy resins [7]. However, strong van der Waals forces exist between GO sheets, inducing the aggregation of GO sheets and causing an uneven stress concentration in epoxy matrix [8]. This can seriously limit the reinforcing effect of GO in epoxy resins. Additionally only a few active groups on GO surface can react with epoxy matrix, resulting in weak interfacial interactions between GO sheets and matrix. These problems can be addressed by functionalizing GO with some small molecules or polymers that can introduce some organic groups such as epoxide groups, amino groups, and isocyanate groups [9,10,11]. For example, Wan et al. [12] reported a modification method of GO by covalent grafting of diglycidyl ether of bisphenol A chains. The surface functionalization was found to obviously improve the compatibility and dispersibility of GO sheets in epoxy matrix, with the tensile strength and fracture toughness increased by 75% and 41% at 0.5 wt % filler loading, compared to the neat epoxy. Ryu et al. [13] prepared the hexamethylene diamine functionalized graphene oxide successfully. The modification could significantly improve the dispersibility of GO sheets and interfacial interactions with the matrix, leading to promote the curing reaction well.

Ionic liquids have attracted a great deal of attention for their unique properties, such as excellent chemical and thermal stabilities, good electrical conductivity, and high ionic mobility [14,15]. Yang et al. [16] synthesized polydisperse graphene nanosheets stabilized by amine-terminated ionic liquids, which could be dispersed well in water, *N*,*N*-dimethylformamide (DMF), and dimethyl sulphoxide (DMSO). They thought the good dispersibility was attributed to the improved solubility and electrostatic repulsion of modified graphene nanosheets because of the ionic liquid incorporation. Due to the good dispersibility provided by ionic liquids, we think that the ionic liquid modified graphene or graphene oxide is of great value for reinforcing polymers. However, most reports about ionic liquid modified graphene or graphene oxide just focus on the electrochemical applications, such as electrodes, sensors, and supercapacitors [17,18]. We think it is necessary to investigate the reinforcing effects of ionic liquid modified graphene or graphene oxide in epoxy resin.

Meanwhile, it is worth mentioning that a majority of ionic liquids contain imidazole groups. As is well known, the imidazole rings can obviously catalyze the curing reactions of epoxy resins [19]. Pour et al. [20] prepared the poly (vinyl imidazole) grafted GO nanosheets and found that imidazole modified GO could enhance the curing rate by decreasing the activation energy of epoxy-amine curing system. Consequently, it might be a good choice to modify GO sheets by imidazolium ionic liquid, in order to enhance the dispersibility of GO sheets, as well as introduce imidazole groups to the epoxy curing system. In this way, it is possible to prepare a multifunctional nanoparticle with catalytic and reinforcing effects in epoxy resins.

Herein, we fabricated the imidazolium ionic liquid (IMD-Si) modified graphene oxide (IMD-Si@GO) by a cheap and simple method, based on the silanization reaction. The IMD-Si@GO nanosheets were incorporated into an epoxy resin-anhydride system to prepare the novel epoxy composites with high performances. The dispersibility of IMD-Si@GO, and the reinforcing and catalytic effects of IMD-Si@GO in the epoxy resin-anhydride system, have been investigated. Additionally, the thermal conductivities and thermal stabilities of IMD-Si@GO/epoxy (IMD-Si@GO/EP) composites were studied.

## 2. Materials and Methods

### 2.1. Materials

Diglycidyl ether of bisphenol A (DGEBA) epoxy with an epoxy value of 0.48–0.54 mol/100 g was purchased from Wuxi Resin Factory of Bluestar New Chemical Materials Co., Ltd., Wuxi, China. Methyltetrahydrophthalic anhydride (MTHPA, Puyang Huicheng Chemicals Co., Ltd., Puyang, China) was used as the curing agent. GO sheets were prepared from natural graphite flakes (500 mesh, Qingdao Hensen Graphite Co., Ltd., Qingdao, China) by a modified Hummers method [21]. Imidazole and 3-chloromethoxypropylsilane were purchased from (Alading Reagent Co., Ltd., Shanghai, China). Ethyl acetate, anhydrous alcohol and acetone were purchased from (Tianjin Tianda Chemical Co., Ltd., Tianjin, China).

### 2.2. Synthesis of IMD-Si@GO

**Scheme 1:**
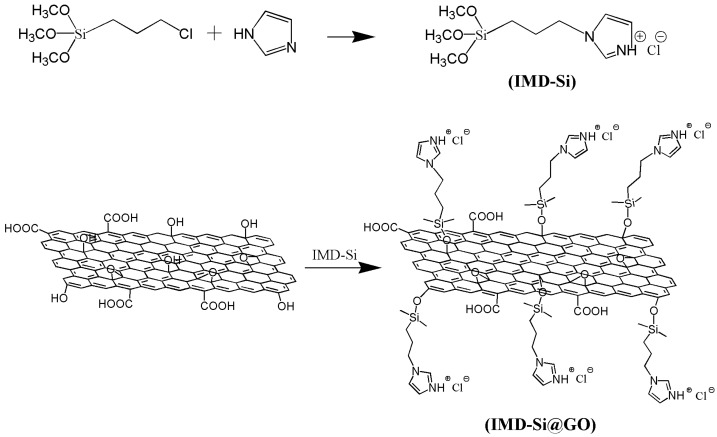
Schematic of synthesis of the IMD-Si@GO.

A schematic representation of the synthesis of IMD-Si@GO is shown in Scheme 1. In the first step, 5.5 mL (30 mmol) 3-chloromethoxypropylsilane and 2.04 g (30 mmol) imidazole were mixed at 110 °C with magnetic stirring for 24 h under N_2_ atmosphere [22]. The ionic liquid was washed by ethyl acetate for several times for purification. Then 500 mg GO was dispersed into 250 mL anhydrous alcohol by sonication for 30 min. IMD-Si (0.5 g) was added into the dispersion purged with N_2_ at 50 °C. After refluxing for 4 h, the mixture was filtered, washed with anhydrous alcohol, and dried in a vacuum oven at 60 °C.

### 2.3. Fabrication of Epoxy Composites

The IMD-Si@GO/EP were prepared by casting method with different contents of fillers (0.1, 0.2, 0.4, 0.6, and 0.8 wt %). The IMD-Si@GO sheets were first dispersed in acetone by sonication and then mixed with DGEBA epoxy. Afterwards, the mixture was put into a vacuum oven at 70 °C to remove solvent. Then, a stoichiometric amount of the curing agent MTHPA was added to the above mixture, followed by stirring for 10 min. Finally, the mixture was poured into a mold, cured following the schedule of 140 °C/2 h + 160 °C/4 h + 180 °C/3 h. As a reference, the pure epoxy resin was also prepared in similar procedures to perform contrast experiments.

### 2.4. Characterization

^1^H Nuclear magnetic resonance (^1^H NMR, 400 MHz) spectra were recorded in deuterated DMSO solvent using a Bruker Avance spectrometer (Bruker Instrument, Billerica, MA, USA). Atomic force microscope (AFM) images were taken in a tapping mode using Hitachi Nanonavi E-sweep (Hitachi High-Tech Science Co., Tokyo, Japan) with the size of 6 μm × 6 μm. X-ray photoelectron spectra (XPS) were performed on a PHI Quantum 2000 Scanning ESCA Microprobe system (ULVAC-PHI Inc., Kanagawa, Japan). All XPS spectra were corrected using the C 1s line at 284.8 eV. Fourier transform infrared (FTIR) spectra were characterized by a PerkinElmer-283B FTIR (Perkin Elmer Inc., Waltham, MA, USA) spectrometer ranging from 4000 to 400 cm^−1^. Elemental analysis was carried out with a VarioEL III Analyser (Elementar Analysensyteme GmbH, Hanau, Germany). X-ray diffraction (XRD) patterns were recorded with a Bruker D8 ADVANCE X-ray diffractometer (Bruker AXS GmbH, Karlsruhe, Germany) (Cu Kα radiation, 0.1542 nm). The XRD data were collected from 5° to 85° with the scanning rate of 0.02°/s. Thermogravimetric analysis (TGA) was performed on a TGA Q50 (TA Instrument, New Castle, DE, USA) at a heating rate of 10 °C/min, in an argon atmosphere.

The dispersion of IMD-Si@GO in epoxy matrix was verified by using transmission optical microscopy (TOM, Pudan MM-8, Pu Dan Optical Instrument Co., Ltd., Shanghai, China). Bending tests and impact tests were carried out in a universal testing machine (CMT-6303, Shenzhen SANS Testing Machine Co., Ltd., Shenzhen, China) following the Chinese standard GB/T2567-2008, and at least five tests were carried out for each sample. The samples’ dimensions for bending tests and impact tests were (80 ± 0.2) × (15 ± 0.2) × (4 ± 0.2) mm^3^ and (80 ± 0.2) × (10 ± 0.2) × (4 ± 0.2) mm^3^, respectively. The fractured surface of epoxy composites obtained from the impact test was observed using a scanning electronic microscope (SEM, Hitachi S-570, Hitachi High-Tech Science Co., Tokyo, Japan) and the samples were sputter-coated with gold. Differential scanning calorimetry (DSC) was conducted on a TA Instruments DSC 2920 (TA Instrument, New Castle, DE, USA) using N_2_ as a purge gas from 30 to 300 °C with a heating rate of 10 °C/min. Thermal conductivities of the samples were measured at room temperature by a Hot Disk instrument (AB Co., Uppsala, Sweden).

## 3. Results and Discussion

### 3.1. Characterization of the IMD-Si@GO

**Figure 1:**
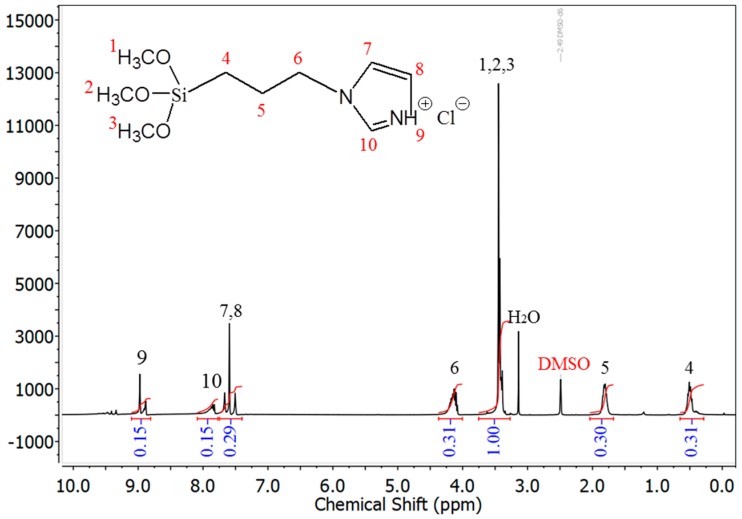
^1^H NMR spectra of IMD-Si.

The chemical structure of IMD-Si was characterized by ^1^H NMR (Figure 1). The H1, H2, and H3 related to methoxyl group, are observed at 3.53–3.38 ppm. The signals of the protons linked to –CH_2_–CH_2_–CH_2_– at 0.61–0.47, 1.91–1.77, and 4.25–4.08 ppm, are respectively corresponding to H4, H5, and H6. The protons marked by 7 and 8, 10 and 9, corresponding to protons in the imidazole ring observed at 7.72–7.50, 7.95–7.83, and 9.03–8.89 ppm, respectively. Additionally, a sharp peak can be seen at 3.29 ppm, which is most likely to correlate to moisture in IMD-Si. It is notable that the peak at 9.03–8.89 ppm shifts to lower field significantly, for H9 is an active hydrogen.

**Figure 2:**
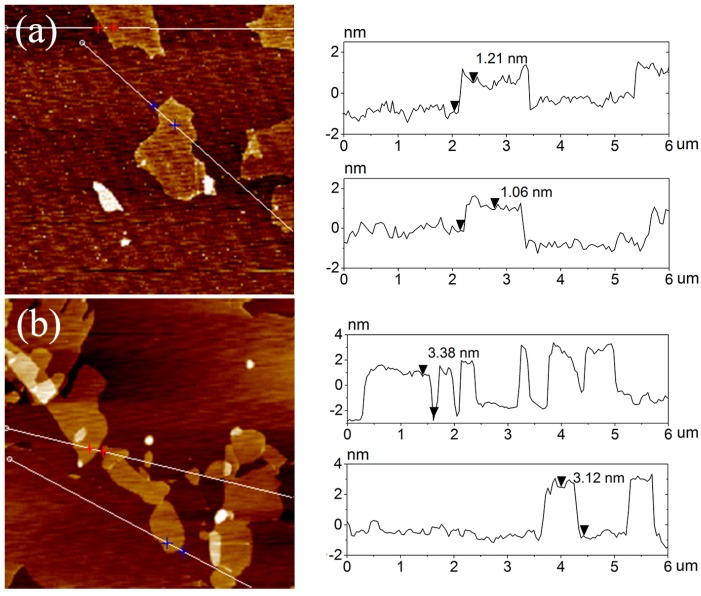
AFM images of GO (**a**) and IMD-Si@GO (**b**).

AFM measurement was used to observe the morphology of GO and IMD-Si@GO, after deposition on mica surface. It can be seen from Figure 2 that single layer GO and IMD-Si@GO sheets of varying size are deposed on the substrates with overlaps. The single layer GO sheets are varied in the thickness range of 1.0–1.3 nm, which is in agreement with the values reported previously [23]. After functionalized by ionic liquid, the thicknesses of single layer sheets are obviously increased to nearly 2.8–3.8 nm, indicating the successful presence of the ionic liquid grafted onto GO sheets.

**Figure 3:**
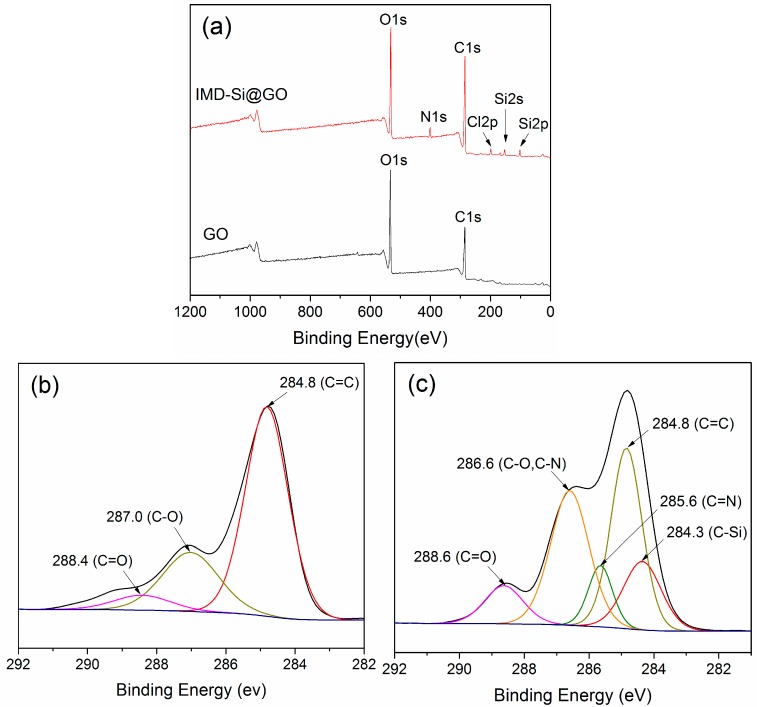
XPS spectra of GO and IMD-Si@GO: (**a**) survey spectra; (**b**) C 1s of GO; (**c**) C 1s of IMD-Si@GO.

XPS spectra are very efficient to characterize the chemical composition and electronic structure of nanomaterials. Figure 3a shows the XPS survey spectra of GO and IMD-Si@GO. As for GO, there are two obvious peaks at 284.8 and 531.8 eV, corresponding to C 1s and O 1s, respectively. After functionalization, new peaks appear at 101.8, 152.8, 198.8, and 400.8 eV attributed to Si 2p, Si 2s, Cl 1s, and N 1s, respectively. The high resolution C 1s XPS spectrum of GO (Figure 3b) shows three types of carbon, located at 284.8 (C=C), 287.0 (C–O), and 288.4 eV (C=O) [24]. Compared with GO, the C 1s band of IMD-Si@GO (Figure 3c) can be fitted to five components: 284.3 (C–Si), 284.8 (C=C), 285.6 (C=N), 286.6 (C–O, C–N), and 288.6 eV (C=O). All these proofs can demonstrate the imidazolium ionic liquid grafted onto GO sheets successfully.

**Figure 4:**
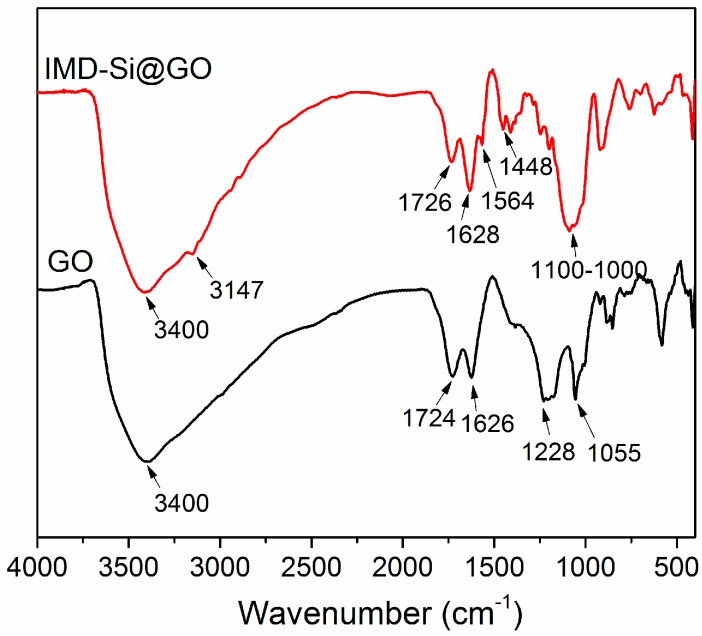
FTIR spectra of GO and IMD-Si@GO.

The FTIR spectra of GO and IMD-Si@GO are shown in Figure 4, which are consistent with XPS data. In the spectrum of GO, the absorption peaks at 3400, 1724, 1626, 1228, and 1055 cm^−1^ are attributed to –OH, –COOH, C=C, epoxy C–O, and alkoxy C–O, respectively [9]. As for IMD-Si@GO, a new peak appears at 3147 cm^−1^ due to the stretching vibrations of C–H in imidazole rings. The bands at 1564 and 1448 cm^−1^ are assigned to C–N and C=N, indicating the imidazole rings introduced onto the GO surface. In addition, a broad absorption band is observed in the range of 1100–1000 cm^−1^ related to Si–O–C and Si–O–Si, which also confirms the presence of imidazolium ionic liquid.

From the XPS and FTIR spectra, it can be concluded that the imidazolium ionic liquid has been successfully grafted onto GO surface. As we know, elemental analysis is an efficient method to calculate the grafting ratio of carbon materials [25]. The carbon, hydrogen, and nitrogen content of IMD-Si@GO are listed in Table 1. The grafting ratio of ionic liquid is defined as the weight percent of IMD-Si to IMD-Si@GO, which is calculated as the nitrogen weight content of IMD-Si@GO divided by that of IMD-Si (calc. 12.6%, not including three methyl groups). The grafting ratio of IMD-Si is calculated to be about 22.8%, that is, the content of IMD-Si groups is 1.03 mmol/g in IMD-Si@GO.

**Figure 5:**
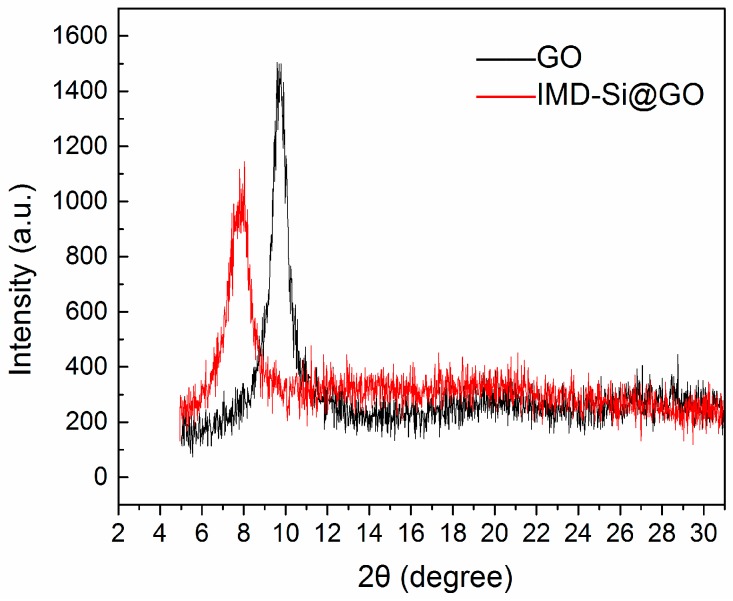
XRD patterns of GO and IMD-Si@GO.

XRD patterns of GO and IMD-Si@GO are showed in Figure 5. The sharp peak at 9.68° for GO corresponds to an interlayer space of 0.91 nm. After functionalization, the diffraction peak shifts to 8.10°, indicating the interlayer distance of 1.09 nm, which can confirm the intercalation of GO sheets by imidazolium ionic liquid. The intercalation of GO makes it easy to dispersed in polymer matrix and prevents the aggregation efficiently, providing a good potential to GO for the preparation of polymer composites [26,27].

**Figure 6:**
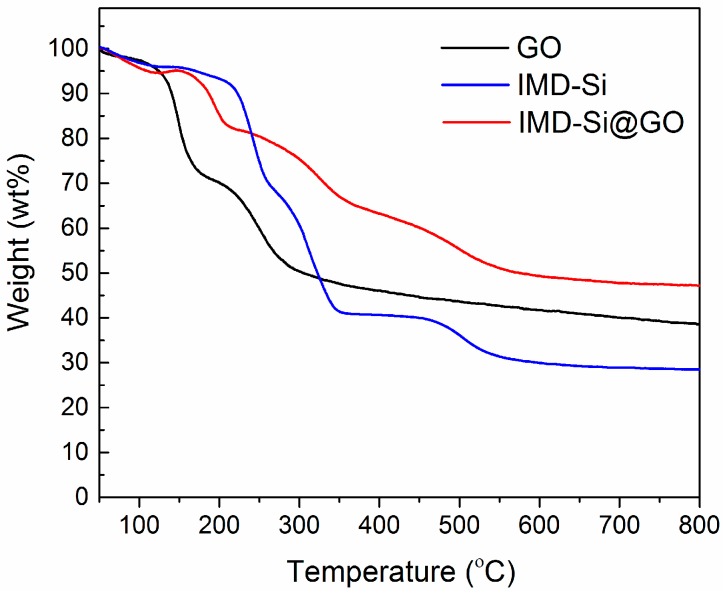
TGA curves of IMD-Si, GO and IMD-Si@GO.

We can see the TGA curves of IMD-Si, GO and IMD-Si@GO from Figure 6. GO has a slight mass loss below 100 °C because of the water evaporation. The main weight reduction for GO at the range of 100–310 °C is 47.7%, attributed to the pyrolysis of the oxygenated groups [28]. As for IMD-Si@GO, the mass loss at 100–310 °C (21.9%) is much lower than that for GO, indicating an enhancement in thermal stability. The weight reduction of IMD-Si@GO is mainly attributed to the pyrolysis of residual oxygenated groups and the decomposition of surface-attached ionic liquid [29]. Additionally the residual weight of IMD-Si, GO and IMD-Si@GO at 800 °C is 28.2%, 38.5%, and 47.1%, respectively. It is worth mentioning that the residual weight of IMD-Si@GO is bigger than that of ionic liquid or GO sheets, also demonstrating the improvement of thermal stability by the grafted ionic liquid. The reason may be that the imidazolium ionic liquid could generate a large amount of Si–O–Si bonds at high temperature, which could greatly hinder the thermal decomposition. In addition, the introduction of imidazole rings onto the GO surface can also slow down the rate of thermal decomposition.

### 3.2. Dispersion and Exfoliation of IMD-Si@GO

**Figure 7:**
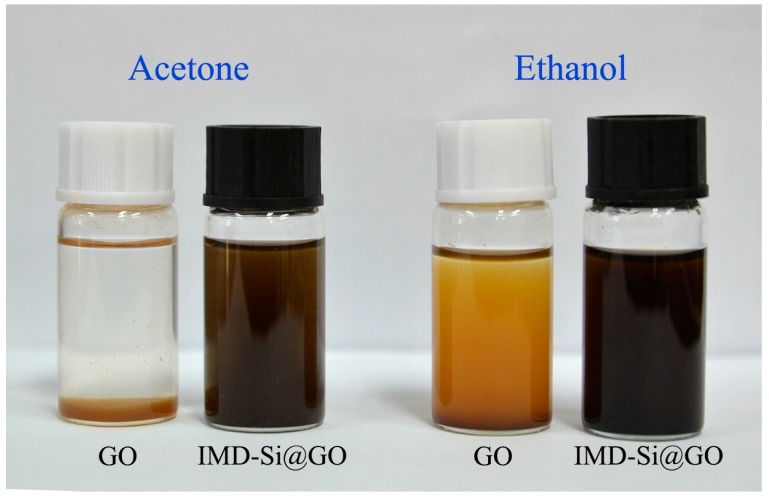
Photos of GO and IMD-Si@GO dispersions in acetone and ethanol.

To test the dispersibility of GO and IMD-Si@GO in organic solvents, both of them were dispersed in acetone and ethanol with a concentration of 0.5 mg/mL by sonication for 10 min. Figure 7 shows the photos of GO and IMD-Si@GO dispersions in acetone and ethanol after storage for 2 h. By way of comparison, the unmodified graphene oxide sheets have a complete settlement in acetone and a significant settlement in ethanol, while the IMD-Si@GO dispersions are completely stable in both solvents. This verifies that covalent grafting of imidazolium ionic liquid can significantly facilitate the dispersibility of graphene oxide, due to the intercalation of GO sheets by ionic liquid and the electrostatic repulsion provided by IMD-Si. The good dispersibility of modified GO sheets in organic solvents makes it easy to fabricate nanomaterials/epoxy composites by solution blending technique, and also provides the potential for various applications.

**Figure 8:**
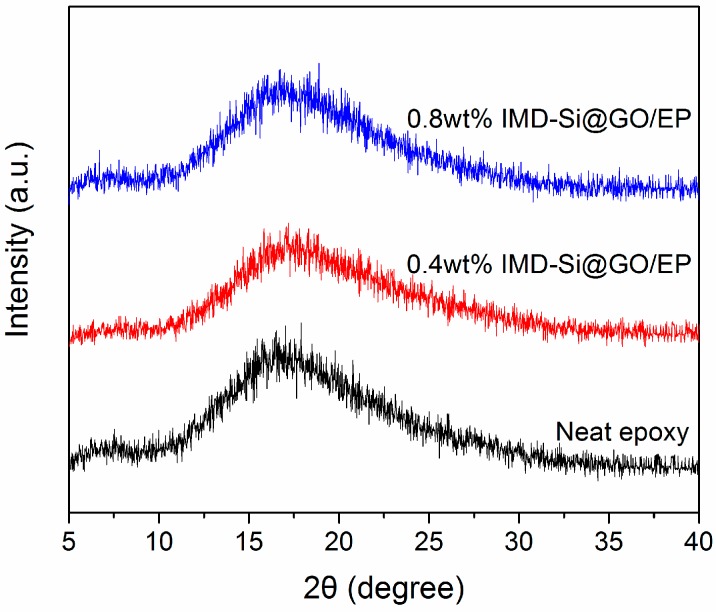
XRD patterns of epoxy composites with different contents of IMD-Si@GO fillers.

The exfoliation degree of the IMD-Si@GO sheets in epoxy matrix was studied by XRD. Figure 8 shows XRD patterns of neat epoxy and the epoxy composites containing 0.4 and 0.8 wt % of fillers. Obviously, there is no apparent difference in terms of the broad diffraction peaks for all these nanocomposites from 11° to 28°. The wide diffraction originates from the scattering of cured epoxy resin. Furthermore, the characteristic diffraction peak of IMD-Si@GO at 8.1° does not appear in both XRD curves of the IMD-Si@GO/EP composites, indicating that IMD-Si@GO nanosheets are well exfoliated in epoxy matrix [12]. It is worth mentioning that good exfoliation degree of nano-fillers does not mean uniform dispersion in matrix, which needs to be further confirmed by TOM photos of cured epoxy resin and SEM photos of the fracture surfaces.

**Figure 9:**
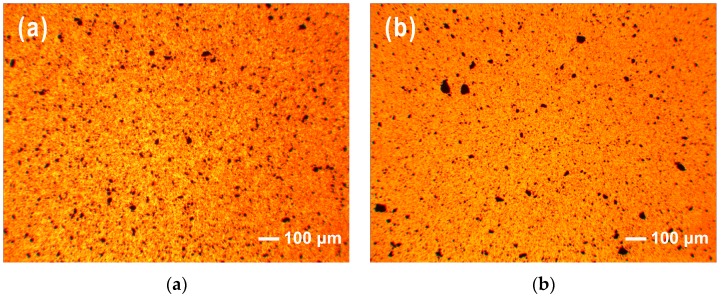
TOM images of epoxy composites with different contents of fillers: (**a**) 0.4 wt %; and (**b**) 0.8 wt %.

In order to evaluate the dispersibility of IMD-Si@GO sheets in epoxy matrix, TOM images of cured epoxy composites with 0.4 and 0.8 wt % fillers were carried out first. As shown in Figure 9a, the IMD-Si@GO sheets present a good dispersion in the matrix at 0.4 wt % filler content, although some small aggregations can been observed. When the filler content is increased to 0.8 wt %, some big clusters of IMD-Si@GO sheets can be easily found. These obvious aggregations may produce stress concentration regions in epoxy matrix and lead to decreased mechanical properties.

### 3.3. Mechanical Properties of IMD-Si@GO/EP

**Figure 10:**
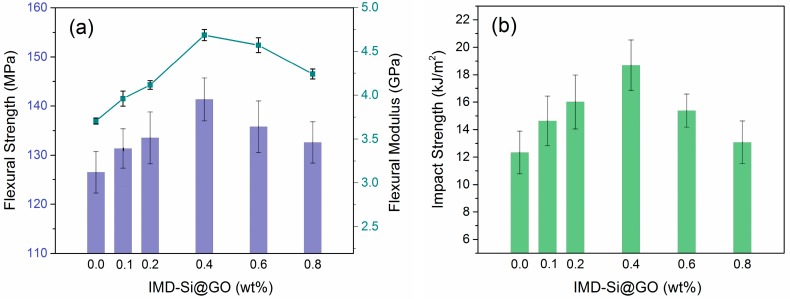
Flexural strength, flexural modulus (**a**) and impact strength (**b**) of epoxy composites with different contents of IMD-Si@GO fillers.

The mechanical properties of epoxy resin with different contents of IMD-Si@GO fillers were experimentally studied. The flexural strength, flexural modulus, and impact strength of IMD-Si@GO/EP composites are shown in Figure 10. It is interesting that the flexural and impact strength of epoxy composites reach the maximum values of 141.3 MPa and 18.7 kJ/m^2^ at 0.4 wt % IMD-Si@GO, increased by 12% and 52% compared with the neat epoxy (126.5 MPa and 12.3 kJ/m^2^), respectively. Additionally it shows a maximum increase of 26% in flexural modulus (4.69 GPa) for epoxy composites containing 0.4 wt % IMD-Si@GO. The enhanced strength and modulus of epoxy by adding a small amount of IMD-Si@GO is considered to be due to the strong interfacial bonding between fillers and epoxy matrix. The imidazole rings and residual oxygenated groups on the IMD-Si@GO surface can participate in the curing reaction. Consequently, the IMD-Si@GO is not only a nano-filler, but also a co-curing agent in epoxy matrix, which can strongly improve the interfacial interactions [30]. Meanwhile, in the presence of IMD-Si, GO sheets can be well exfoliated and uniformly dispersed in the matrix, promoting the reinforcing effect, obviously [31]. By increasing the filler content continuously, the flexural strength, flexural modulus and impact strength starts to decrease. This may impute to the following two points: (i) too high a filler loading induces the aggregation of IMD-Si@GO sheets in the matrix, which can be seen in Figure 9b, leading to weakening of the mechanical properties of epoxy composites; and (ii) too much IMD-Si@GO hinders the curing reaction and may decrease the crosslinking density [32,33].

**Figure 11:**
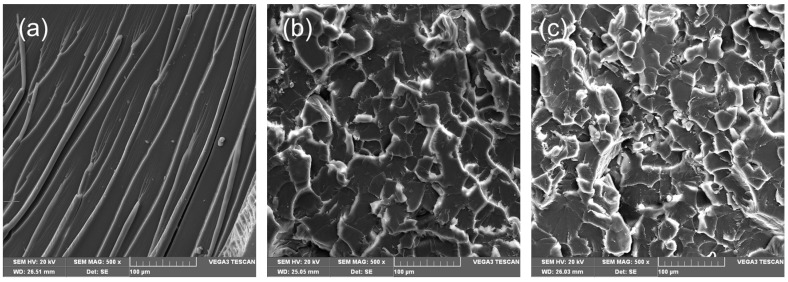
SEM images of the fracture surfaces of epoxy composites with different contents of IMD-Si@GO fillers: (**a**) neat epoxy; (**b**) 0.4 wt %; (**c**) 0.8 wt %.

To further study the reinforcing mechanism of IMD-Si@GO in epoxy matrix, the fracture surfaces of epoxy and its composites were investigated by SEM. In Figure 11a, the neat epoxy specimen shows a river-like fracture surface, indicating the characteristics of brittle fracture without stress dispersion [34]. Figure 11b shows a rough and uneven fracture surface of 0.4 wt % IMD-Si@GO/EP, exhibiting a typical tough feature. This proves a significant enhancement of the interfacial adhesion between the epoxy matrix and fillers [35]. In addition, almost no aggregation of IMD-Si@GO sheets can be seen on the surface, demonstrating a good dispersibility of IMD-Si@GO in the epoxy matrix. When the filler content is increased to 0.8 wt %, some aggregations can be found on the fracture surface from Figure 11c, which is in good agreement with the observation of TOM images and analysis of the mechanical properties.

### 3.4. Catalytic Effect of IMD-Si@GO in Epoxy Resin-Anhydride System

**Figure 12:**
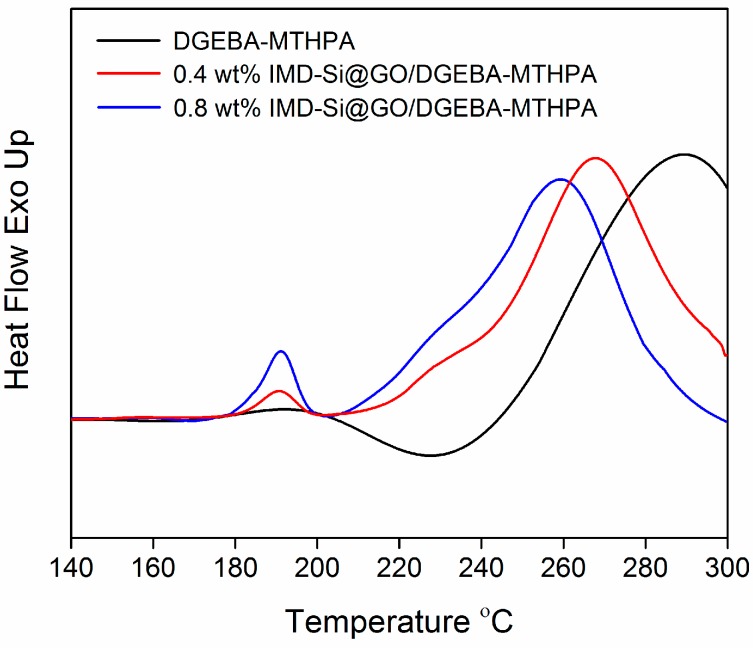
DSC curing curves of epoxy resin-anhydride with different contents of IMD-Si@GO with a heating rate of 10 °C/min.

Imidazole and its derivatives are widely used as a type of accelerators in epoxy resin-anhydride systems, which can initiate alternating copolymerization between anhydride and epoxy groups, as a result, initiate the esterification reaction and improve the curing speed dramatically. Thence, the catalytic effect of IMD-Si@GO in epoxy resin-anhydride system was investigated by DSC (Figure 12). For the neat DGEBA-MTHPA curing system, a broad exothermic peak with a peak temperature at 289 °C has been seen for a heating rate of 10 °C/min. As for DGEBA-MTHPA with 0.4 wt % IMD-Si@GO, the exothermic peak moves to around 268 °C and becomes sharper. By increasing the filler content to 0.8 wt %, the broad peak continues to shift to the left with a peak temperature at 259 °C. This indicates that imidazole modified GO can indeed play the role in catalyzing epoxy resin-anhydride curing system, and the increase in the content of IMD-Si@GO results in a higher reaction rate [36,37]. Additionally it is obvious that a small peak appears at around 191 °C by adding IMD-Si@GO nanosheets, without movement when the filler content rises. This peak may originate from the reaction between the pyridine nitrogen and epoxy groups.

**Scheme 2:**
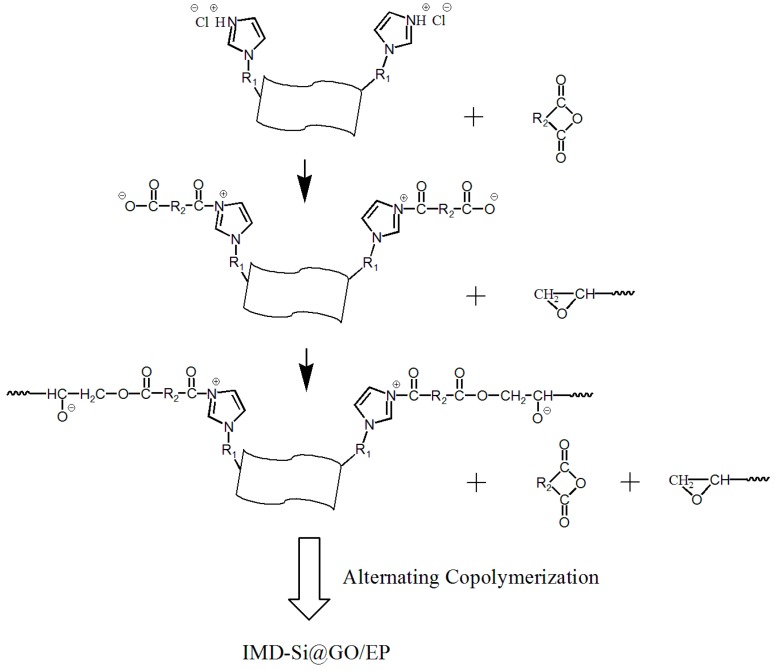
Catalytic mechanism of IMD-Si@GO/epoxy resin-anhydride system.

The main catalytic mechanism of IMD-Si@GO in epoxy resin-anhydride system is anionic ring-opening alternating copolymerization, which is shown in Scheme 2. The imidazole rings on the IMD-Si@GO surface attack anhydride groups to generate carboxylate anions. Then the carboxylate anions initiate the ring-opening reaction to yield oxygen anions. Oxygen anions on the IMD-Si@GO surface subsequently transform to carboxylate anions by reacting with other anhydride groups. Thence, alternating copolymerization continues to take place between the anhydride and epoxy groups to form cross-linked polymer networks [38]. It can be concluded that the IMD-Si@GO can initiate esterification reaction, that is to say, the functionalized graphene oxide can be a catalyst in epoxy resin-anhydride systems.

### 3.5. Thermal Conductivities of IMD-Si@GO/EP

**Figure 13:**
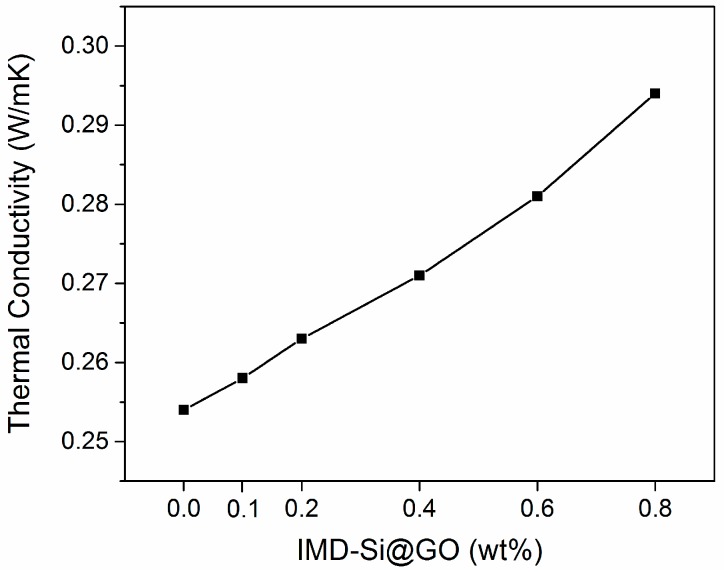
Thermal conductivities of epoxy composites with different contents of IMD-Si@GO fillers.

Thermal conductivity is also effectively affected by carbon fillers within the epoxy matrices [39]. Figure 13 shows the thermal conductivities of the IMD-Si@GO/EP composites prepared with 0.1, 0.2, 0.4, 0.6 and 0.8 wt % of fillers. We can see that the thermal conductivities of IMD-Si@GO/EP composites are increased with increased contents of fillers. The thermal conductivity is greatly improved to 0.294 W/mK with only a little amount of IMD-Si@GO (0.8 wt %), getting a 15.7% increase compared to that of unfilled epoxy (0.244 W/mK) [40]. From the enhancement of thermal conductivities, we can deduce at least following two points: (i) there are strong interfacial interactions between epoxy and IMD-Si@GO sheets, which could reduce the thermal interfacial resistance effectively; and (ii) the uniform dispersion of IMD-Si@GO in epoxy matrix results in an increased contact area between IMD-Si@GO and matrix and promotes the phonon diffusion efficiency [41].

### 3.6. Thermal Stabilities of IMD-Si@GO/EP

**Figure 14:**
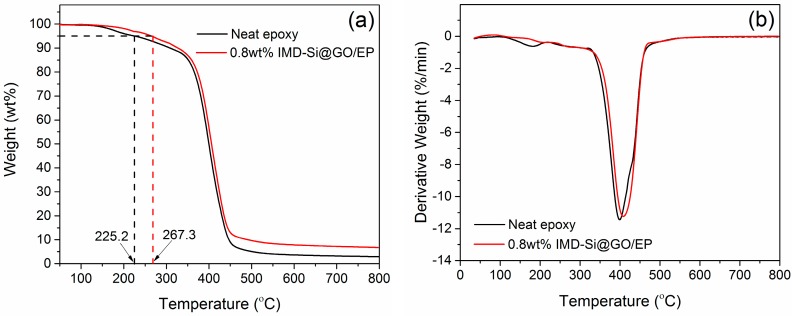
TGA (**a**) and DTG (**b**) curves of the neat epoxy and 0.8 wt % IMD-Si@GO/EP composites.

Thermal stabilities of the neat epoxy and IMD-Si@GO/EP composites loading 0.8 wt % of nano-fillers were investigated by TGA analysis. TGA and derivative thermogravimetry (DTG) curves are shown in Figure 14. The neat epoxy has *T*_d_ (the temperatures at 5 wt % weight loss) and *T*_max_ (the temperatures at maximum weight loss) of 225.2 and 399.0 °C, respectively. By adding 0.8 wt % IMD-Si@GO, the *T*_d_ and *T*_max_ (267.3 and 408.1 °C) are both higher than these of neat epoxy, suggesting that IMD-Si@GO can efficiently inhibit the mass loss during thermal degradation process. As for the char yield at 800 °C, the char residue of 0.8 wt % IMD-Si@GO/EP dramatically increases to 6.41 wt %, 2.2 times compared to that of unfilled epoxy (2.91 wt %). The enhancement of thermal stabilities can be explained by the barrier effect of modified GO sheets, uniform dispersion of IMD-Si@GO in matrix, and also strong interfacial bonding between IMD-Si@GO sheets and epoxy matrix [42].

## 4. Conclusions

In this study, imidazolium ionic liquid modified graphene oxide was prepared by a cheap and simple method. The results from AFM, XPS, FTIR, XRD, and TGA indicate the successful preparation of IMD-Si@GO, and the grafting ratio is calculated to be 22.7% from the elemental analysis. The interlayer spacing of GO sheets is increased by intercalation of IMD-Si, which strongly facilitates the dispersibility of graphene oxide in organic solvents and epoxy matrix. The flexural strength, flexural modulus and impact strength of the IMD-Si@GO/EP composites are optimal with 0.4 wt % fillers, increased by 12%, 26%, and 52%, respectively, compared to the neat epoxy. Additionally, DSC reveals that the IMD-Si@GO sheets can catalyze the curing reaction of epoxy resin-anhydride system at very low IMD-Si@GO loadings. Moreover, a little inclusion of IMD-Si@GO into epoxy matrix can remarkably improve the thermal conductivities and thermal stabilities. These enhancements can be attributed to the uniform dispersion of IMD-Si@GO and strong interfacial interactions with the epoxy matrix. Consequently, the modification of GO sheets by covalent grafting of ionic liquid could be a promising method in the application of reinforced polymers by nanomaterials.

## Figures and Tables

**Table 1:** Elemental analysis for IMD-Si@GO.

Element name	C	H	N
IMD-Si@GO (wt %)	43.64	3.07	2.87
